# Blow Collection as a Non-Invasive Method for Measuring Cortisol in the Beluga (*Delphinapterus leucas*)

**DOI:** 10.1371/journal.pone.0114062

**Published:** 2014-12-02

**Authors:** Laura A. Thompson, Tracey R. Spoon, Caroline E. C. Goertz, Roderick C. Hobbs, Tracy A. Romano

**Affiliations:** 1 Mystic Aquarium, a division of Sea Research Foundation, Mystic, Connecticut, United States of America; 2 Marine Sciences Department of the University of Connecticut, Avery Point, Groton, Connecticut, United States of America; 3 Alaska Sea Life Center, Seward, Alaska, United States of America; 4 National Marine Mammal Laboratory, Alaska Fisheries Science Center, Washington, United States of America; ETH Zurich, Switzerland

## Abstract

Non-invasive sampling techniques are increasingly being used to monitor glucocorticoids, such as cortisol, as indicators of stressor load and fitness in zoo and wildlife conservation, research and medicine. For cetaceans, exhaled breath condensate (blow) provides a unique sampling matrix for such purposes. The purpose of this work was to develop an appropriate collection methodology and validate the use of a commercially available EIA for measuring cortisol in blow samples collected from belugas (*Delphinapterus leucas*). Nitex membrane stretched over a petri dish provided the optimal method for collecting blow. A commercially available cortisol EIA for measuring human cortisol (detection limit 35 pg ml^−1^) was adapted and validated for beluga cortisol using tests of parallelism, accuracy and recovery. Blow samples were collected from aquarium belugas during monthly health checks and during out of water examination, as well as from wild belugas. Two aquarium belugas showed increased blow cortisol between baseline samples and 30 minutes out of water (Baseline, 0.21 and 0.04 µg dl^−1^; 30 minutes, 0.95 and 0.14 µg dl^−1^). Six wild belugas also showed increases in blow cortisol between pre and post 1.5 hour examination (Pre 0.03, 0.23, 0.13, 0.19, 0.13, 0.04 µg dl^−1^, Post 0.60, 0.31, 0.36, 0.24, 0.14, 0.16 µg dl^−1^). Though this methodology needs further investigation, this study suggests that blow sampling is a good candidate for non-invasive monitoring of cortisol in belugas. It can be collected from both wild and aquarium animals efficiently for the purposes of health monitoring and research, and may ultimately be useful in obtaining data on wild populations, including endangered species, which are difficult to handle directly.

## Introduction

Glucocorticoids, such as cortisol, provide important information for wildlife health and conservation efforts; serving as indices of fitness and stressor load as well as for monitoring the response of individuals or populations to stressors [1], [2]. Physiological consequences of increased cortisol, which include reallocation of energy resources [1], [3], inhibition of growth, reproduction and immune function [1], [3], [4] may be beneficial or pathological and ultimately affect the fitness of an individual.

Typical methodology for measuring changes in stress hormones involves blood sampling. Animals in zoos or aquaria, such as marine mammals, can be trained to participate in behavioral blood draws; however, for free ranging animals the process of chase, capture and restraint are stressors in themselves and the data need to be interpreted with consideration of confounding effects of sampling procedures. For some free-ranging species, such as large baleen whales which cannot be restrained or handled, blood sampling is simply not feasible. Alternative matrices for steroid hormone measurements have been successfully collected for both terrestrial and marine animals, including feces, urine, hair, [2], [5], [6], feathers [7] and blubber in marine mammals [8], [9], [10]. While these sampling procedures may be less invasive than blood sampling, they pose their own challenges. For example, there is a risk of environmental contamination when collecting feces or urine from cetaceans. Additionally, the hormone content in these matrices may accumulate over different time periods (e.g. days to months) and thus may be difficult to associate with any particular event or stressor. Use of these matrices need to be evaluated for individual research applications.

Over the past few years, exhaled respiratory condensate or “blow” has been a focus for non-invasive biological sampling in order to monitor steroid hormones, such as testosterone and progesterone in bottlenose dolphins (*Tursiops truncatus*) [11], humpback whales (*Megaptera novaeangliae*) and north Atlantic right whales (*Eubalaena glacialis*) [5], as well as for monitoring bacterial communities [12] and genetics [13]. Exhalations are composed not only of gas and water vapor, but also molecular aerosols, lung mucosa and associated proteins [14], [15]. Cortisol has been measured in exhaled breath condensate from healthy bottlenose dolphins, including a pregnant female [16] and humpback whales (*Megaptera novaeangliae*) [17]. Hunt *et al.*, [18] measured several steroid hormones, including cortisol, in blow collected from north Atlantic right whales.

Methodologies for measuring cortisol in blow samples include radio-immunoassay (RIA) and enzyme-immunoassay (EIA) [19], as well as high performance liquid chromatography coupled with mass spectrometry (HPLC-MS) [5], [11]. HPLC-MS requires expensive specialized equipment and training, while RIA's require the use of radioisotopes [2]. Commercially available EIA's are more portable for field work, and do not require expensive equipment or radioisotopes and therefore provide a more convenient method for monitoring hormones in blow of marine mammals at zoos and aquaria.

The purpose of this work was to evaluate the use of blow as an alternative matrix for monitoring cortisol levels in belugas (*Delphinapterus leucas*). To do this we 1) developed an appropriate sampling protocol for collecting blow in beluga whales 2) validated a commercially available enzyme immunoassay for measuring cortisol in whale blow and 3) measured cortisol content of blow and blood in aquarium and wild belugas before, during and following events known to stimulate activity of the hypothalamic pituitary adrenal (HPA) axis.

## Methods

### Ethics Statement

Paired blow and blood samples were obtained utilizing positive behavioral reinforcement from four beluga whales, two females (∼30 years old) and two males (∼9 and 26 years old), housed at the Mystic Aquarium, Mystic, CT. Paired samples were also collected from wild belugas during live capture-release health assessments in Bristol Bay, AK (n = 15) and Point Lay, AK (n = 1). This study was approved by the Mystic Aquarium Institutional Animal Care and Use Committee and constitutes protocol # 11001. Wild beluga sampling occurred under National Marine Fisheries Services Marine Mammal Research Permit No. 14245 and Alaska Department Fish and Game Permit No. 14610.

### Preliminary Experiments

#### Determination of Collection Device and Material for Blow

Preliminary experiments were carried out using samples collected only from aquarium animals. The volume of blow collected was measured for 3 different collection devices; 50 ml conical tubes, 250 ml Nalgene bottles, and Petri dishes (100 mm×15 mm). Petri dishes consistently returned the largest volumes of blow and were chosen as the collection device for all further work.

Petri dishes were covered with one of four different collection materials; cotton gauze, nylon stocking cleaned by sonication for 20 minutes [5], nitex membrane (110 µm pore size) (Elko Filtering Co., Miami, FL) and tulle netting (Michaels Stores, Waterford, CT), both cleaned with 70% ethanol dehydrant. Each collection material was held in place on the petri dish with an elastic band.

**Figure 1:**
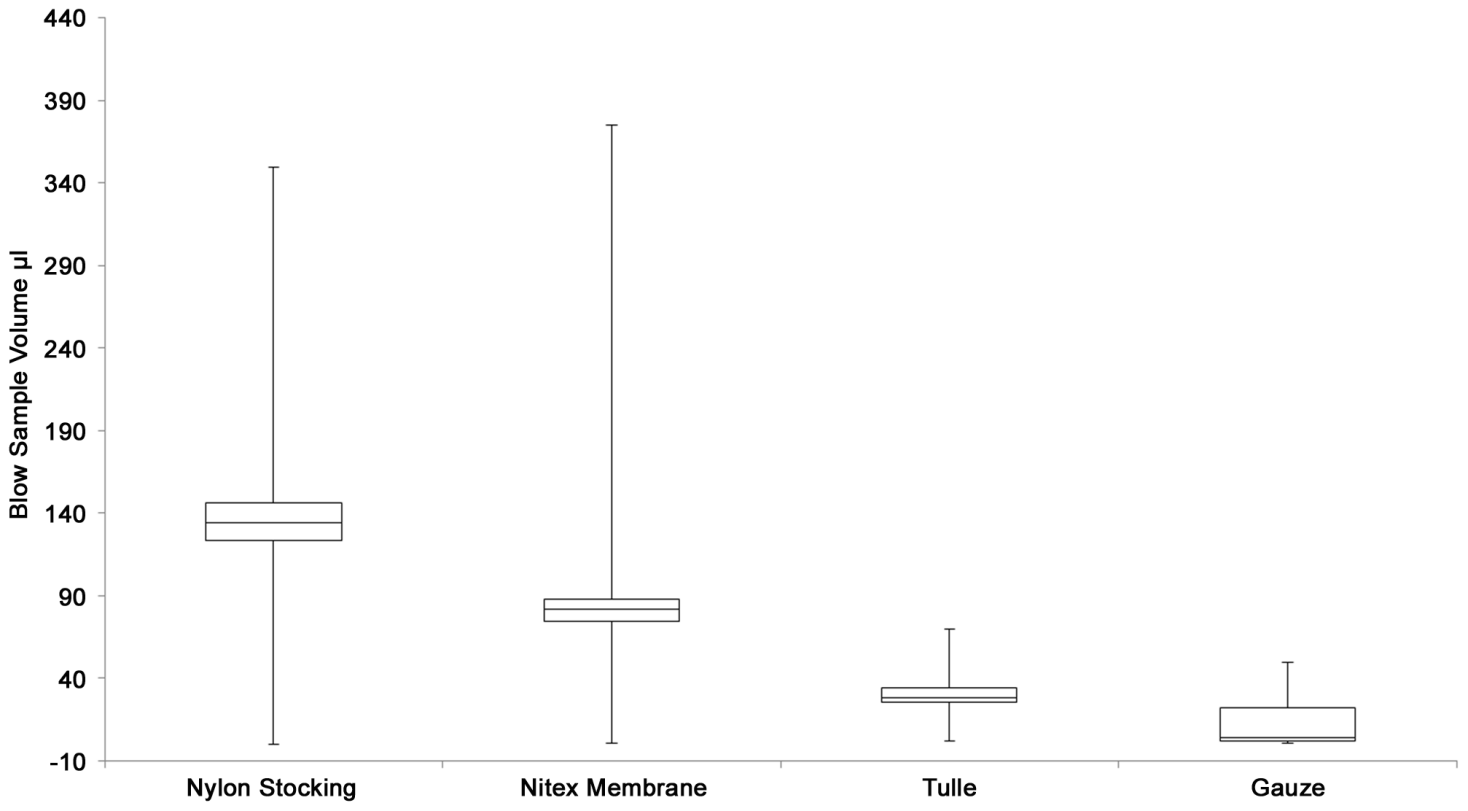
Volumes of blow samples recovered from nylon stocking, nitex (nylon) membrane, tulle netting and gauze following 4 exhales. For each collection material sample collection data are pooled for three animals. Largest volumes were collected from nylon stocking and nitex membrane.

On average ≤30 µl of sample were recovered from cotton gauze and tulle, while ≥50 µl were recovered from nylon stocking and nitex membrane (Figure 1).

**Figure 2:**
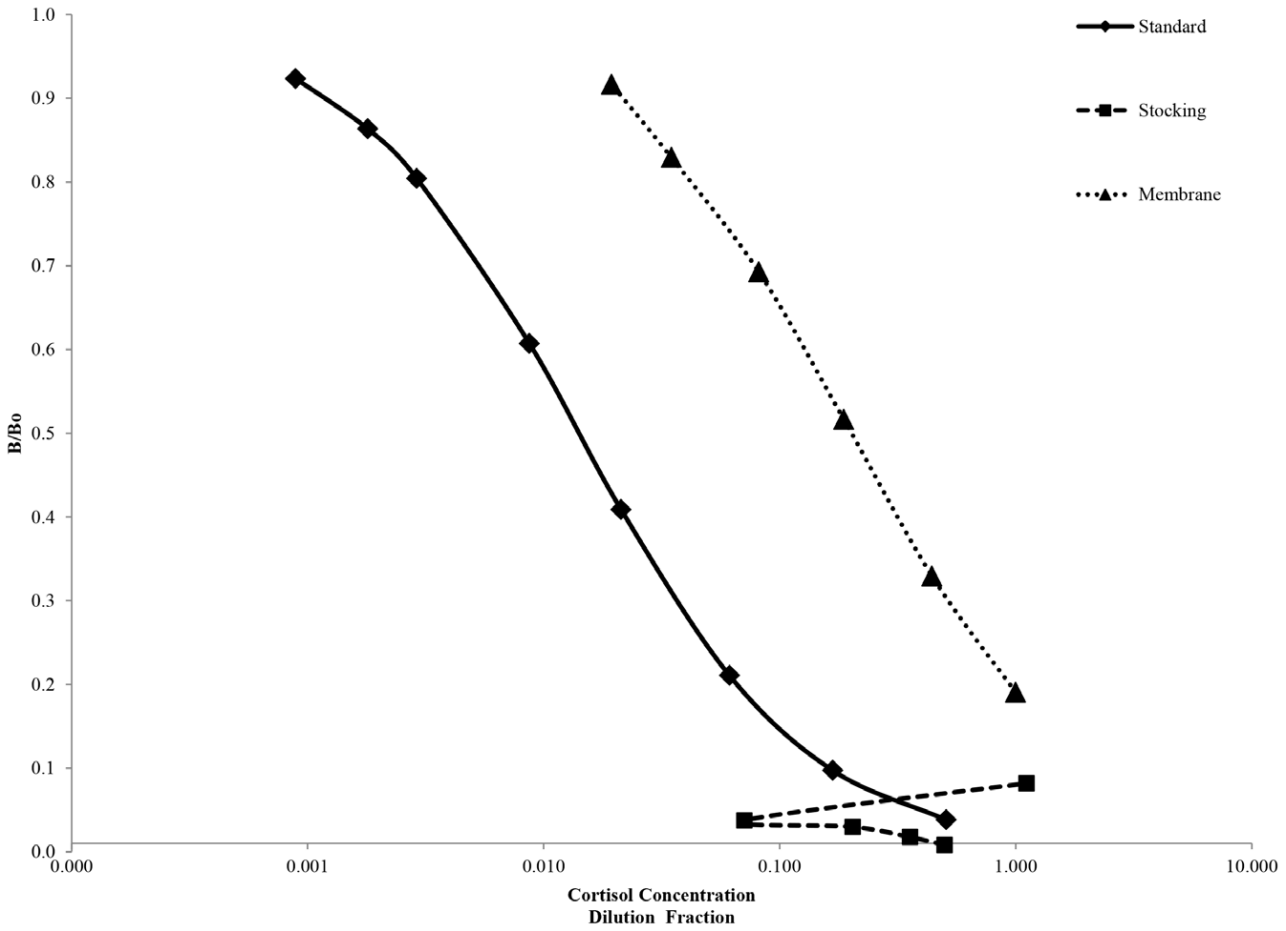
A representative dilution series showing parallelism between cortisol standard with blow samples recovered from nylon stocking and nitex membrane. Nitex membrane shows good parallelism while nylon stocking displays a deviated pattern.

Preliminary experiments to determine the best collection material were run using an EIA from Cayman Chemical (Ann Arbor, MI; cat#500360) which has previously been utilized and validated for marine mammal samples at the Mystic Aquarium. Results (Figure 2) showed parallelism for the nitex membrane with the standard curve whereas results for the nylon stocking suggest less suitability for use. Nitex membrane covered petri dishes were then chosen as the collection device and material for the remainder of the study.

#### Determination of Enzyme Immunoassay

**Figure 3:**
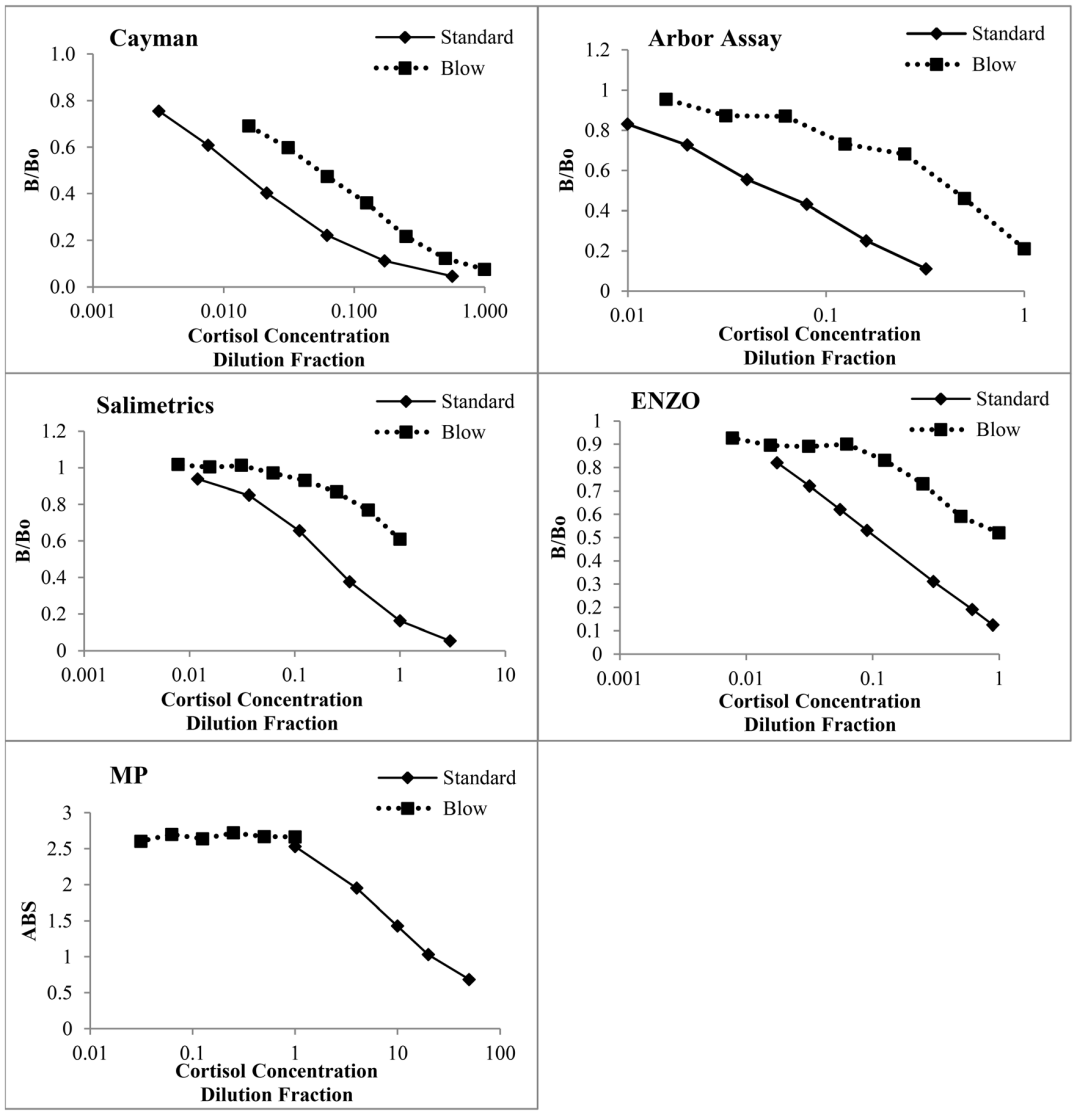
Parallelism results for pooled blow (n = 3) for 5 commercial enzyme-immunoassay's. The B/Bo could not be calculated for MP Biomedical due to differences in kit protocols, therefore absorbance values were used in this case. Cortisol concentration of standards or dilution factor of pooled samples are plotted on the x axes (Log scale).

Immunoassay kits tested for suitability of use with blow, were purchased from Salimetrics (State College, PA; cat#1-3002), MP Biomedical (Solon, OH; cat #07M-21602), Enzo Life Sciences (Miami, FL; cat #ADI-900-071), Cayman Chemical and Arbor Assays (Ann Arbor, MI; cat # K003-HI). Blow samples from three animals (1 male, 2 females) were pooled for kit testing, with a pooled plasma sample run simultaneously as a control. Parallelism results for all kits are shown in figure 3. In addition, the dilution for achieving 50% B/Bo (ratio of sample cortisol bound compared with maximum binding) and the required volume of sample to reach this value was also calculated for each kit (Table 1). Based on a combination of these results, as well as simplicity of methodology and analysis, the Cayman Kit was chosen for all further validation.

**Table 1:** Calculated volumes of blow required per well to reach 50%B/Bo (the middle of the standard curve) for each cortisol EIA tested.

Assay Kit	Vol of unknown required per well (µl)	Dilution for 50% B/Bo	Actual Vol. of sample needed per well (µl)
Cayman	50	1∶4 to 1∶16	3.125–12.5
Arbor Assays	50	1∶2	25
MP Biomedical	25	ND	ND
ENZO	100	1 to 1∶2	50–100
Salimetrics	25	1	25

MP biomedical did not provide measurements necessary to calculate the %B/Bo and so absorbance values were used during assessment (indicated by ND  =  no data).

### Cortisol Enzyme Immunoassay

#### Validation of Cayman Chemical EIA

Blow samples were thawed at room temperature and centrifuged at 2060×g for 15 minutes. This centrifugation step is recommended when working with saliva samples in order to remove mucins which precipitate during freezing [20] and so was tested with blow samples. Pellets were observed in many of the blow samples, particularly those of larger volume. Following centrifugation the supernatant was removed for use in the assay. Blow samples were pooled for males (n = 2) and females (n = 2) separately. All samples were run in duplicate. Parallelism was tested by serially diluting pooled samples 1∶2, 1∶4, 1∶8, 1∶16 and 1∶32. The dilution factor and resulting B/Bo were graphed and compared subjectively with the slope of the standard curve. Intra-assay variability was calculated from eight replications of triplicate wells of a pooled blow sample. Inter-assay variability was calculated using 6 cortisol standards, provided with the kit, between 40 and 4000 pg ml^−1^ and averaging the % CV over three separate assays.

Cortisol standards were also used to test accuracy of cortisol in blow samples, and recovery of cortisol from the collection membrane. For accuracy, four standards (41, 102.4, 256, and 640 pg ml^−1^) were spiked into equal volumes of male and female pools of blow (i.e. 200 µl standard and 200 µl blow pool). The measured cortisol minus cortisol measured in un-spiked samples, was compared with the expected values of the standard.

In addition, 200 µl of three standards (102.4, 256, and 640 pg ml^−1^) were spiked directly onto collection membranes and retrieved following a 30 minute centrifugation at 2800×g for 30 minutes at 10°C (i.e. the same protocol as blow samples) to test for recovery.

Monthly, OWE and wild samples were run in duplicate at a dilution of 1∶4. Where cortisol concentrations were outside the 20–80% B/Bo of the kit, samples were rerun at an adjusted dilution, volume permitting.

Non-specific binding wells (NSB), blanks, and standard curves were run on all plates. The cortisol EIA monoclonal antibody has a reported 100% cross reactivity with cortisol and 4% cross reactivity with prednisolone. In addition <2% cross reactivity is reported with Cortexolone, and <1% cross reactivity with 11-Deoxycorticosterone, 17-hydroxyprogesterone, cortisol glucuronide, corticosterone, cortisone, androstenedione, enterolactone, estrone, 17- hydroxypregnenolone, pregnenolone, and testosterone. Kit sensitivity is 35 pg ml^−1^.

All samples were run in duplicate. Plates were accepted if the maximum binding wells displayed absorbance values between 0.3 and 1.0 with the blanks subtracted. Sample results were accepted if the percent bound fell within the 20–80% range and CV's for duplicates were no larger than 30%.

### Sample Collection

#### Blow Collection and Processing

Based on preliminary experiments petri dishes with nitex membrane were used for all sample collection. Collection dishes were kept refrigerated or on ice for at least 30 minutes prior to sample collection to aid in the condensation of sample, based on the development of collection devices for human breath condensate studies [14], [15].

#### Monthly Values in Aquarium Belugas

The whales at Mystic Aquarium were trained through positive behavioral reinforcement to exhale on signal. For validation and monthly samples, belugas were stationed with their head resting on the exhibit beach. A single exhale was performed first to clear water from the blow hole. The collection device was held inverted approximately 2–4 inches from the blow hole for the duration of 4 or 8 exhales. While 4 deep exhales were found to provide a decent volume of sample during preliminary experiments, the exhales in two animals were short and shallow chuffs, thus 8 exhales were targeted in these animals to increase the volume of sample collection.

Monthly samples were collected from 3 animals (1 male, 2 females) between 2011 and 2013 with the addition of monthly samples from a fourth animal (male) in 2012, in order to monitor cortisol levels throughout the year and begin to investigate seasonal variation in cortisol production. For seasonal analysis, data was grouped in three month bins (e.g. Winter  =  Jan, Feb, Mar; Spring  =  April, May, June; Summer  =  July, Aug, Sept; Fall  =  Oct, Nov, Dec).

#### Out of Water Examination in Aquarium Belugas

“Stressor” samples were obtained from three animals (1 male, 2 females) during out of water examinations (OWE) by attaching petri dishes to one end of a pole extended over the blow hole. For the OWE, animals were lifted out of the water on a stretcher and placed on padding upon the exhibit beach for examination including gross full body exam, blood sampling, and veterinary assessment. Paired blow and blood samples were taken at 10 minute intervals throughout the 30 minute examination. Baseline samples for the OWE occurred following the same protocol as monthly sampling (i.e. under positive behavioral reinforcement) at 1 week and 24 hours prior to the OWE itself.

At the conclusion of the examination, animals were returned to the water and samples were collected at a further 24 hours, 48 hours, 72 hours and 96 hours post OWE. At the end of sample collection, petri dishes were covered and secured with the membrane inside and placed on ice.

#### Wild Belugas

**Figure 4:**
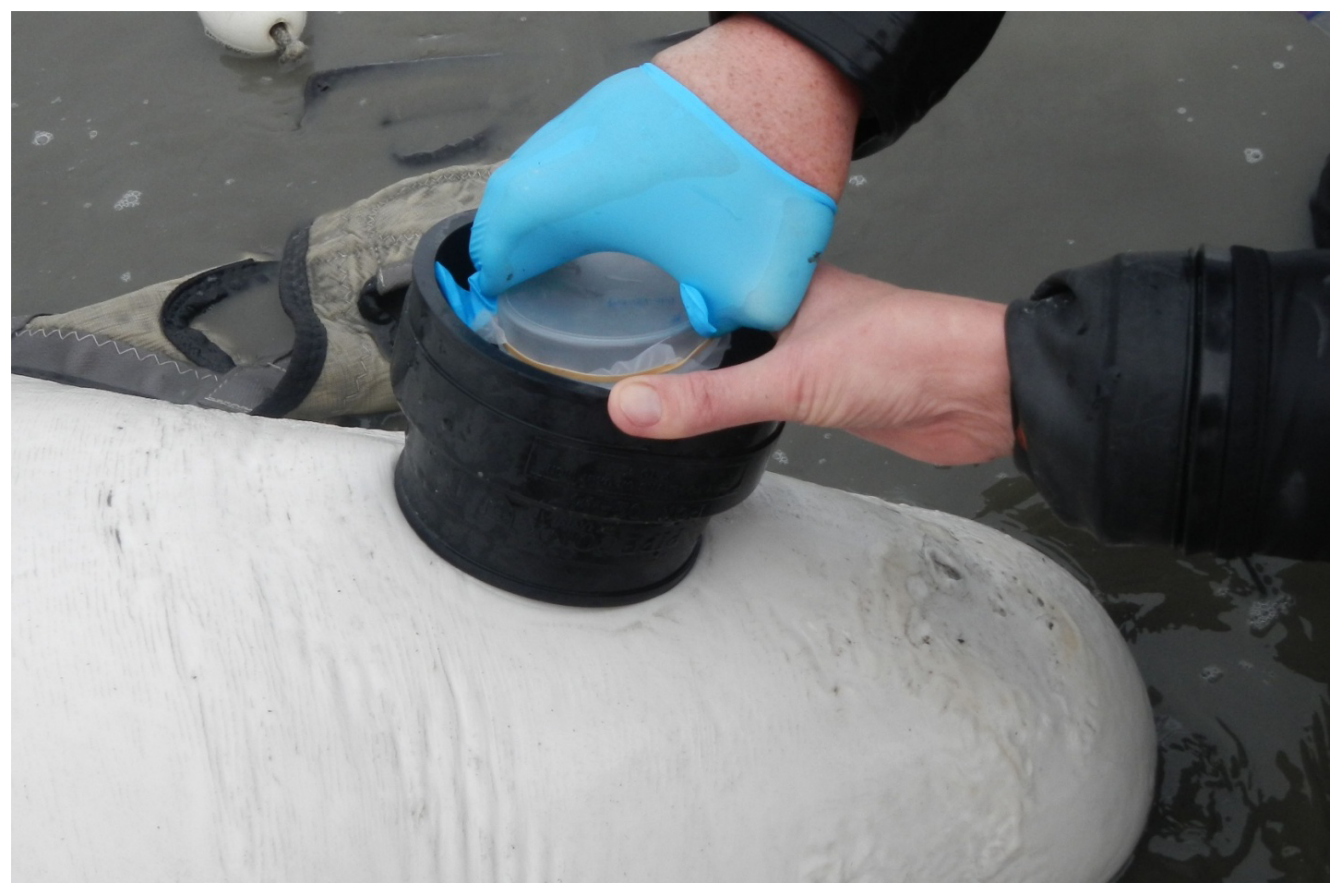
Collection of blow from a wild beluga in Bristol Bay, AK during live-capture and release health assessment studies. A plastic gasket was placed around the blowhole in order to minimize water contamination of blow samples. The blow plate was then held inverted in the center of the gasket for two repeated exhales.

Wild belugas were captured and handled in the fall of 2012 and 2013 according to Norman *et al.*, [21]. Animals were guided into shallow water using several small boats and captured in netting suspended between two boats. For handling, animals were removed from the net and each whale was supported so that the blowhole remained out of the water between breaths. One exhale was allowed to occur before collection in order to clear water from the blowhole. For animals in deeper water, a plastic gasket was placed around the blowhole to aid in keeping water out (Figure 4). Due to conditions of field sampling and animal restraint, samples could not be taken at a consistent time. However an initial sample (‘pre’) was taken in conjunction with a blood draw as soon after entanglement as possible. All pre samples were taken within 45 minutes of hitting the net. Following complete health examination including sampling and tagging, a final sample (‘post’) was also taken. Post sampling occurred 47–93 minutes from entanglement. ‘Post’ blood samples were available for cortisol analysis only during 2013. On average, 2 exhales were collected opportunistically per plate, based on opportunity. Following sample collection, membranes were secured inside petri dishes. Plates were stored inside plastic bags and placed in a cooler with an ice pack until they could be transferred to a −20°C freezer (<10 hours). Plates were then shipped back to the Mystic Aquarium on dry ice.

In the lab, the collection membrane was used to wipe the inside of the dish in order to collect any sample which passed through it, and placed inside a 50 ml conical tube with a plastic stopper made from the sterile plunger of a 20cc syringe. Conical tubes were centrifuged at 2800×g for 30 minutes at 10°C. The centrifuged condensate was then pipetted into 1.25 ml Sarstedt tubes and stored at −80°C to avoid degradation [6] until assay.

#### Blood Collection and Processing

Blood samples were obtained from belugas at the same sessions during which blow samples were collected. Blood was drawn from the ventral or dorsal flukes and collected in 10 ml sodium heparinized vacutainer tubes. Blood tubes were kept on ice until centrifugation at 2000×g for 10 minutes at 10°C. Plasma was aliquoted into 1.25 ml tubes and stored at −80°C. Subsequently, one ml aliquots were sent to the AHDC Endocrinology Laboratory at Cornell University, Ithaca, NY, for cortisol analysis [22], [23] via Immulite chemiluminescent enzyme immunoassay (Siemens Medical Solutions, Malvern, PA, cat # LKC01).

### Statistics

Statistical analysis was conducted using IBM SPSS Statistics Version 21. A Shapiro-Wilk test for normality revealed a significant deviation from normal in data from monthly samples, OWE samples, as well as wild belugas (p<0.001). Following a log transformation, data from monthly samples were normal and a repeated measures ANOVA was used to compare monthly cortisol values. However, a non-parametric Kruskal Wallis ANOVA was required to compare cortisol values over time points during the course of the OWE for aquarium animals, and between ‘pre’ and ‘post’ conditions for Bristol Bay whales.

Because animal populations experiencing any type of stressor show greater variance in physiological responses [24] heterogeneity of variance was also investigated by calculating the absolute differences between each data point and the mean, and comparing baseline, 30 minutes out of water and 24 hours post out of water examination using a non-parametric repeated measures ANOVA.

Correlations were conducted in order to compare changes in blow and plasma cortisol for the OWE's, as well as compare blood and blow cortisol for ‘pre’ samples from Bristol Bay animals. In addition, regression analyses were employed to determine the relationship between blow cortisol and sample volume, as well as between handling time and both blood and blow cortisol in Bristol Bay belugas. For all tests, significance was determined at α = 0.05.

## Results

### Cortisol Kit Validation

Following determination of nitex membrane as the collection material and a collection protocol during preliminary experiments, several commercial cortisol EIA's were tested and the Cayman Chemical cortisol EIA was chosen for further validation based on preliminary parallelism results, and kit requirements. Intra and inter assay CV's were calculated to be 8.26 and 11.79 respectively, both of which fall within the acceptable range for recommended use of the kit.

**Figure 5:**
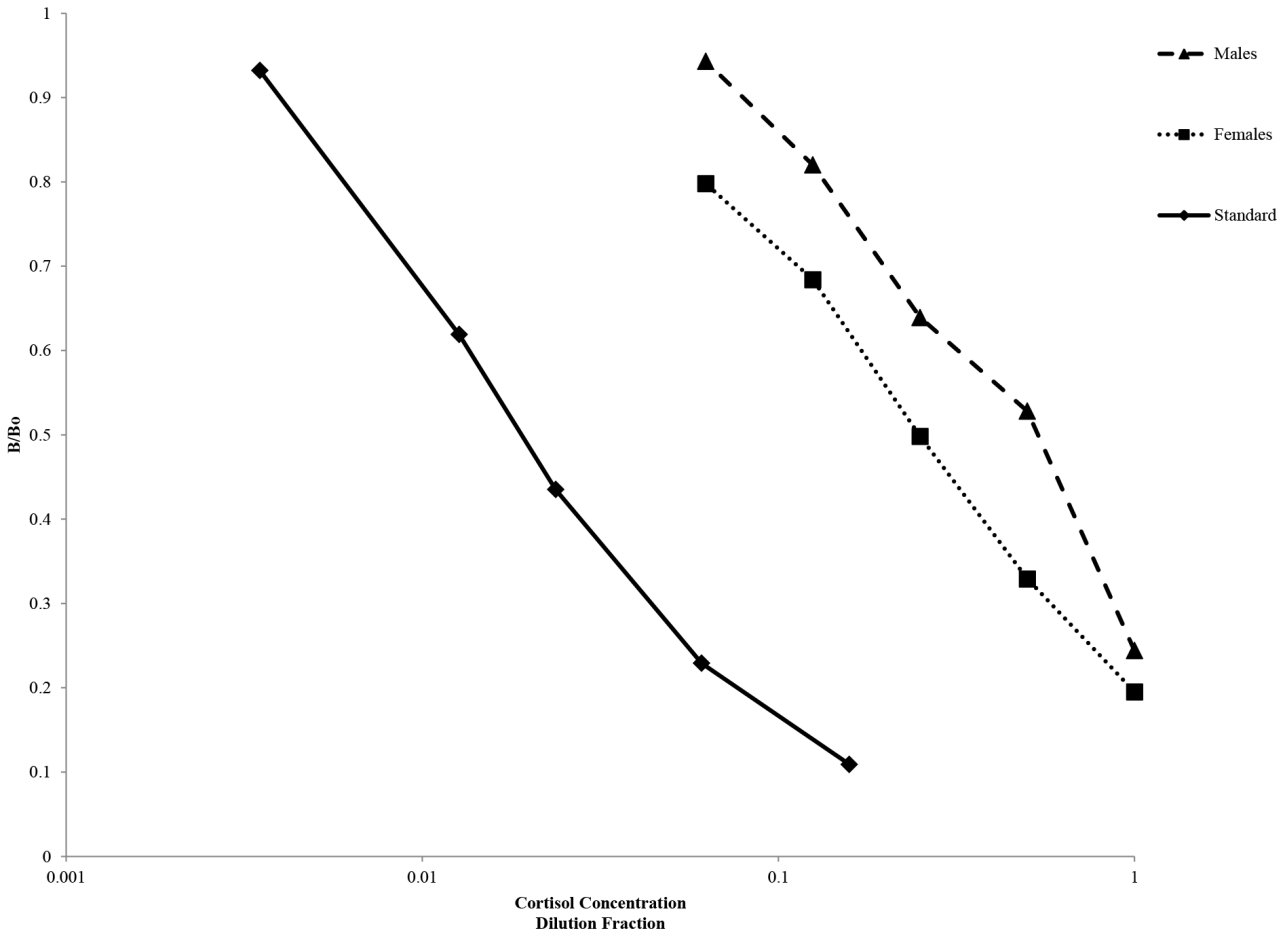
Parallelism results for male and female pools of blow compared with the standard curve. For standards B/Bo is plotted against the concentration of each standard (Log scale) while for the blow pools the B/Bo is plotted against the dilution factor (Log scale).

**Figure 6:**
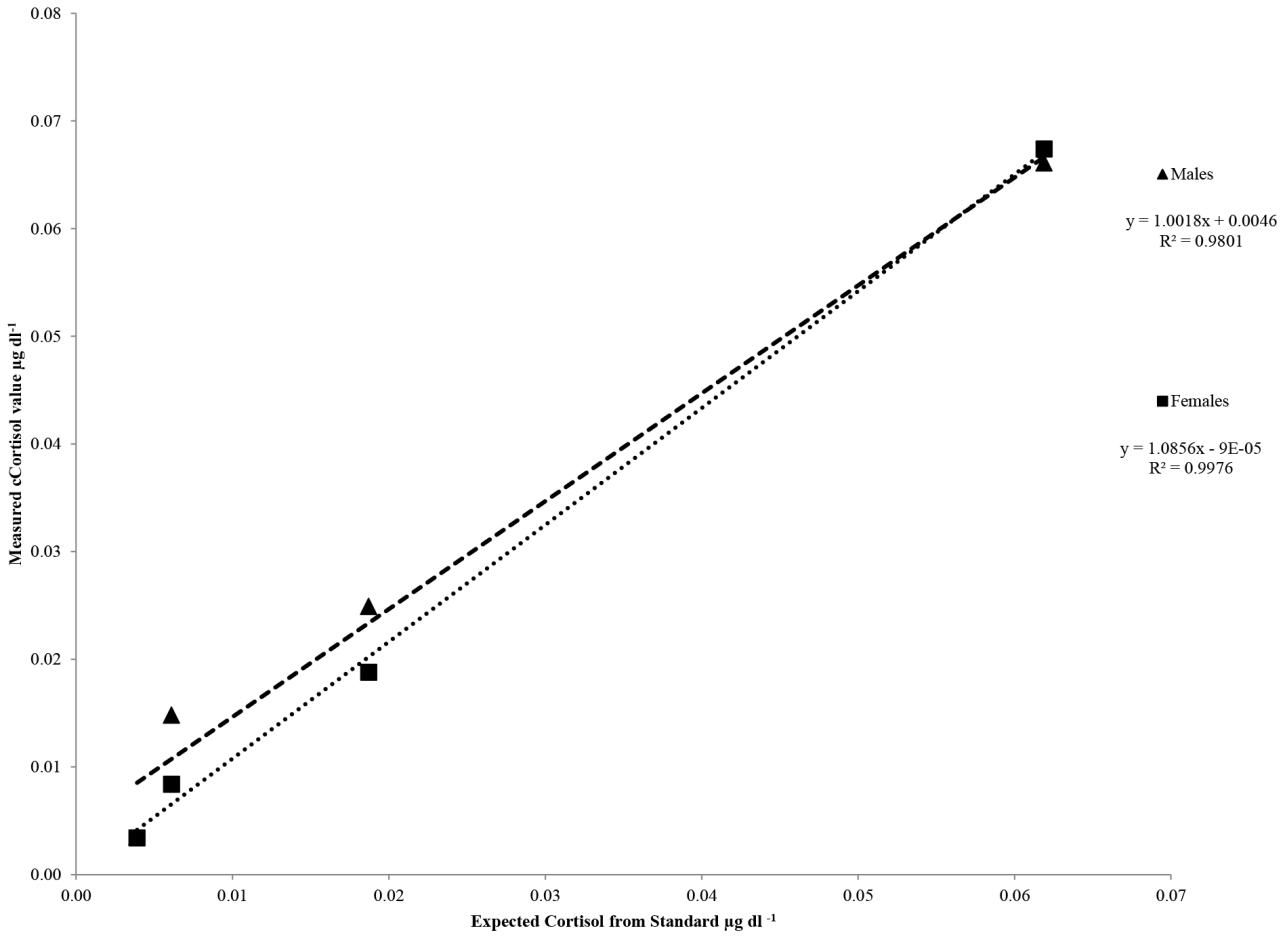
Results of accuracy testing for the Cayman kit using pooled male (blue diamond) and female (red square) blow samples. Measured cortisol values are calculated with the actual sample cortisol subtracted and compared against the known standard concentrations.

Parallelism results were repeated for this kit using separate pools of male and female blow. Both pools displayed curves of parallelism to the standard curve (Figure 5). Pooled blow samples were spiked with four standards (41, 102.4, 256, and 640 pg ml^−1^) and measured values (with the cortisol measured in un-spiked samples subtracted) were compared with the expected values of the standards (Figure 6). Males displayed a slope of 0.9999±0.102 while females displayed a slope of 1.086±0.038. Regression analysis revealed significant relationships for both males and females (α = 0.05) suggesting good accuracy of the kit. Three standards were used to spike nitex membrane (102.4, 256, and 640 pg ml^−1^). Measured cortisol concentrations were between 80 and 110% recovery.

### Monthly Values in Aquarium Belugas

**Figure 7:**
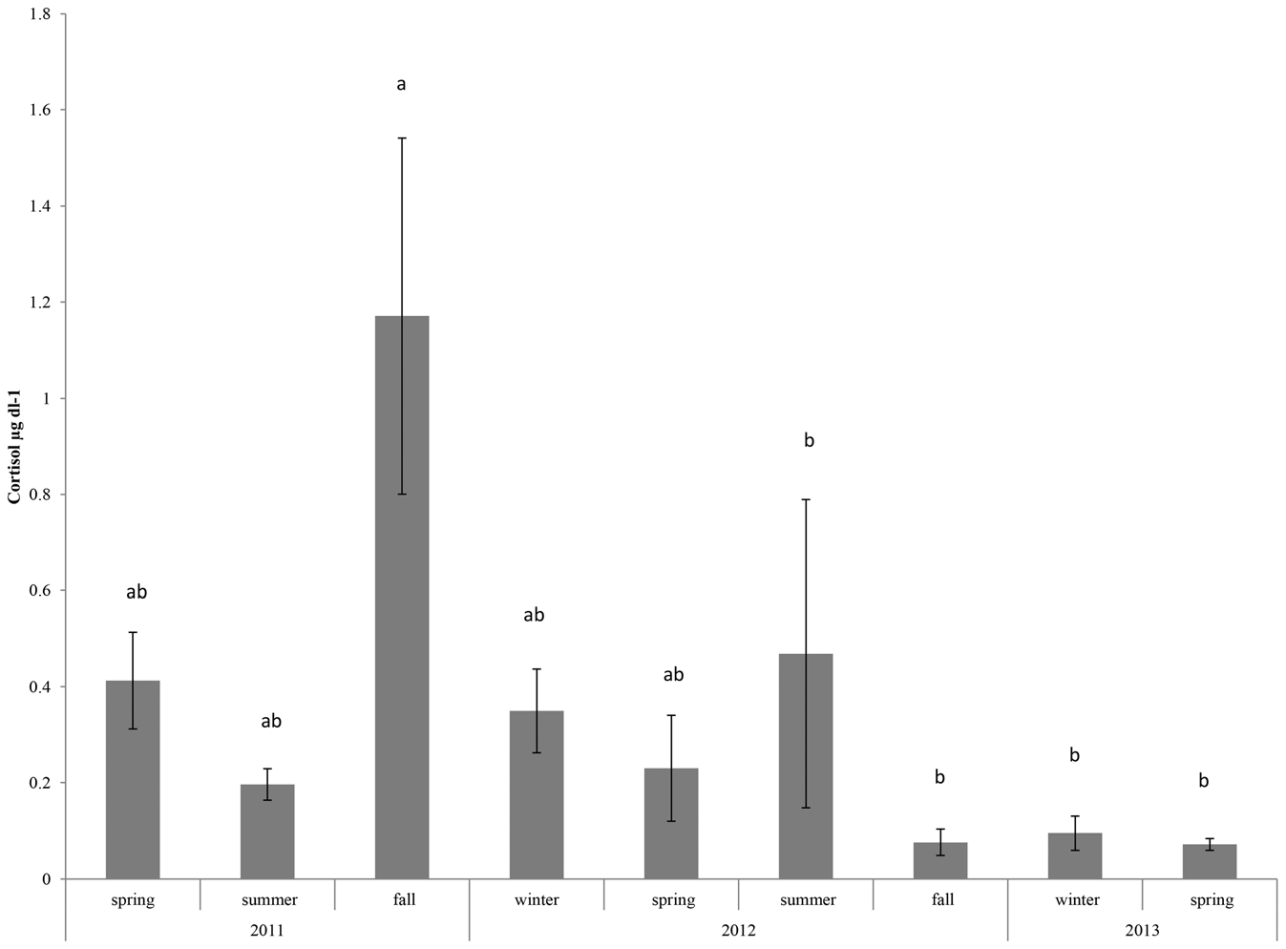
Average cortisol concentrations in blow (± SE) binned by season for 2011, 2012 and 2013. Letters indicate similarities and differences between seasons (i.e. all seasons marked with an ‘a’ are similar, but differ from those marked with a ‘b’). Cortisol values were higher during the fall of 2011.

Cortisol concentration was measured in blow samples collected under positive behavioral reinforcement from four aquarium belugas during monthly health checks between April 2011 and Oct 2013 (Table 2). Missing values reflect samples which ran outside the 20–80% B/Bo range of the cortisol kit or occur where samples were not obtained due to either extreme low volumes (<5 µl) or whale behavior. The majority of blow samples contained less than 1 µg dl^−1^ cortisol (average 0.22 µg dl^−1^), though some variation between months is apparent. No significant differences in monthly cortisol values measured in blow were detected (p>0.05) although increased blow cortisol concentrations were observed in three animals during late summer and fall of 2011, with a peak occurring in Dec 2011. Data were also binned in season by year (winter  =  Jan, Feb, Mar; spring  =  April, May, June; summer  =  July, Aug, Sept; fall  =  Oct, Nov, Dec). Significant differences in cortisol were detected (p = 0.042) though small sample sizes limited the sensitivity of post hoc analyses for detecting changes between specific seasons. Nonetheless, the highest values occurred in the fall of 2011 (Figure 7) with the lowest values occurring in the fall of 2012 through spring of 2013.

**Table 2:** Monthly cortisol values of blow for individual animals between 2011 and 2013.

		Animal1	Animal2	Animal3	Animal4
2011	April	0.7548	1.0470	0.3004	Ø
	May	0.2094	ND	ND	Ø
	June	ND	0.0374	0.1271	Ø
	July	0.1221	I	0.4549	Ø
	August	0.0963	0.0431	0.0474	Ø
	Sept	0.1827	0.4465	0.1276	Ø
	Oct	0.4559	0.5089	I	Ø
	Nov	0.6080	0.5409	0.4819	Ø
	Dec	0.8421	4.6000	1.5080	Ø
2012	Jan	0.7309	0.4999	0.2205	Ø
	Feb	0.1500	0.1109	0.0660	0.8648
	Mar	0.2107	ND	0.1733	0.2862
	April	ND	Ø	ND	Ø
	May	ND	Ø	ND	ND
	June	0.4807	0.1468	Ø	0.0623
	July	0.0970	0.0970	2.5250	0.3979
	August	I	0.0941	0.3343	0.0889
	Sept	I	0.3024	ND	0.0614
	Oct	0.2119	0.0359	ND	0.2650
	Nov	ND	ND	ND	0.0527
	Dec	0.0291	0.0197	0.0313	0.0557
2013	Jan	0.2417	Ø	0.0629	0.0412
	Feb	0.1070	Ø	0.0881	Ø
	March	Ø	Ø	0.0563	Ø
	April	ND	0.0296	0.0216	ND
	May	ND	0.1494	0.0880	Ø
	June	Ø	Ø	Ø	Ø
	July	0.0717	0.1905	Ø	Ø
	August	Ø	Ø	Ø	Ø
	Sept	Ø	0.3054	0.7554	Ø
	Oct	Ø	0.2501	Ø	0.0910

ND  =  not detectable or outside the 20–80% B/Bo range of the Cayman kit. I =  insufficient volume recovered to run sample. Ø =  no sample collected.

### Out of Water Examination in Aquarium Belugas

**Figure 8:**
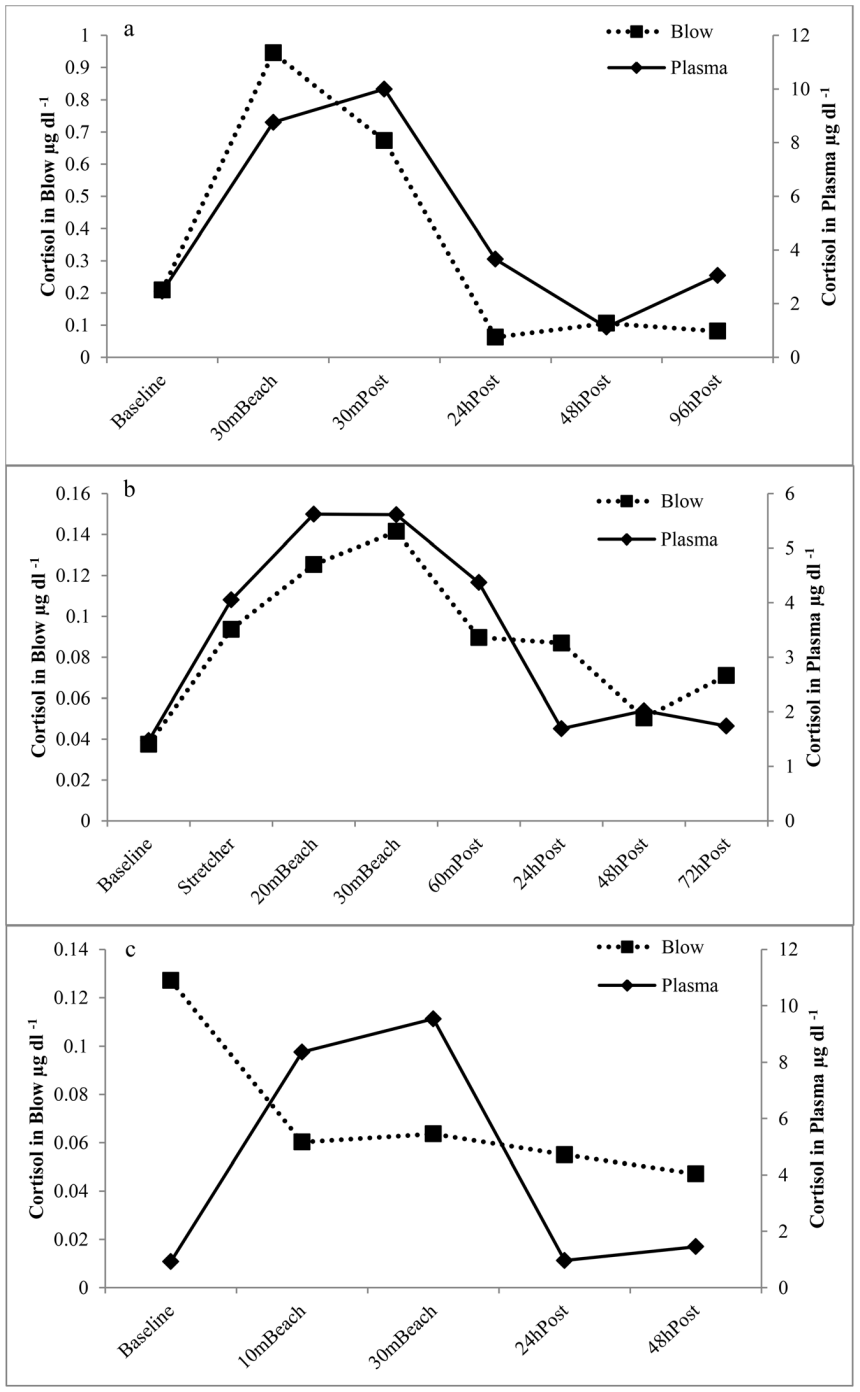
Blow and Plasma cortisol concentrations measured in a) animal 1, b) animal 2, and c) animal 3, over the time course of an OWE. Missing time points reflect samples with volumes too low to run with the assay or where results fell outside the 20–80% B/Bo range.

Breaths for each of the animals involved in the OWE were noted to be shorter and shallower than during most monthly collections. All animals showed significantly (p = 0.039) elevated plasma cortisol at 30 minutes on the beach as compared with baseline samples (with levels returning to baseline by the 24 hours post examination. Animals 1 and 2 also appeared to display increased cortisol in blow at the 30 minute time point though statistical significance was not detected (p>0.05). Heterogeneity of variance for each data point was calculated as the absolute value of the difference between the point and the mean for baseline, 30 min and 24 hour post time points. Heterogeneity of variance was significantly greater for the 30 minute time point as compared to baseline or 24 hour post samples (F = 11.991; p = 0.020). A significant positive correlation was found between blow and plasma cortisol (r = 0.561; p = 0.005). Animals 1 and 2 displayed similar patterns in blow and blood cortisol over the course of OWE 1 while animal 3 did not display any apparent relationship between cortisol in blow and blood (Figure 8).

### Wild Belugas

The volume of blow condensate recovered from one to two exhales per wild beluga ranged from 0 to 800 µl (Table 3). Plates which were found to by dry once returned to the lab were excluded from analysis as no volume of sample could be recovered. For one animal, the same plate was used for both ‘pre’ and ‘post’ sampling and so was removed from analyses for this experiment. In addition, sampling of one animal was exposed to significant amounts of water contamination and was removed. Final analyses for Bristol Bay belugas included 10 ‘pre’ samples and 9 ‘post’ samples. Of these samples, 7 were matched ‘pre’ and ‘post’ for the same animal.

**Table 3:** Volumes and cortisol concentrations of blow samples recovered from live captured belugas in Bristol Bay, AK during 2012 and 2013, and a single animal from Pt. Lay, AK.

	Pre		Post		
Animal ID	Volume µl	Cortisol µg dl^−1^	Volume µl	Cortisol µg dl^−1^	Change
					(Pre to Post)
**DLBB12-01**	Ø	---	10	0.3079	---
**DLBB12-02**	Ø	---	Ø	---	---
**DLBB12-03**	15	0.0311	25	0.5948	Increase
**DLBB12-04**	200	0.2303	90	0.3079	Increase
**DLBB12-05**	25	0.1277	10	0.3636	Increase
**DLBB12-06**	75	0.1947	400	0.2379	Increase
**DLBB12-07**	800	Outside kit range	Ø	---	---
**DLBB12-08**	450	0.0165	800	Outside kit range	---
**DLBB12-09**	300	Outside kit range	Ø	---	---
**DLBB13-03**	Ø	---	50	0.0994	---
**DLBB13-05**	200	0.1651	Ø	---	---
**DLBB13-06**	15	0.1325	50	0.1441	Increase
**DLBB13-07**	50	0.1636	Ø	---	---
**DLBB13-08**	50	0.038	100	0.1646	Increase
**DLBB13-09**	250	0.0163	500	0.0057	Decrease

Ø indicates no sample recovered from membranes.

**Figure 9:**
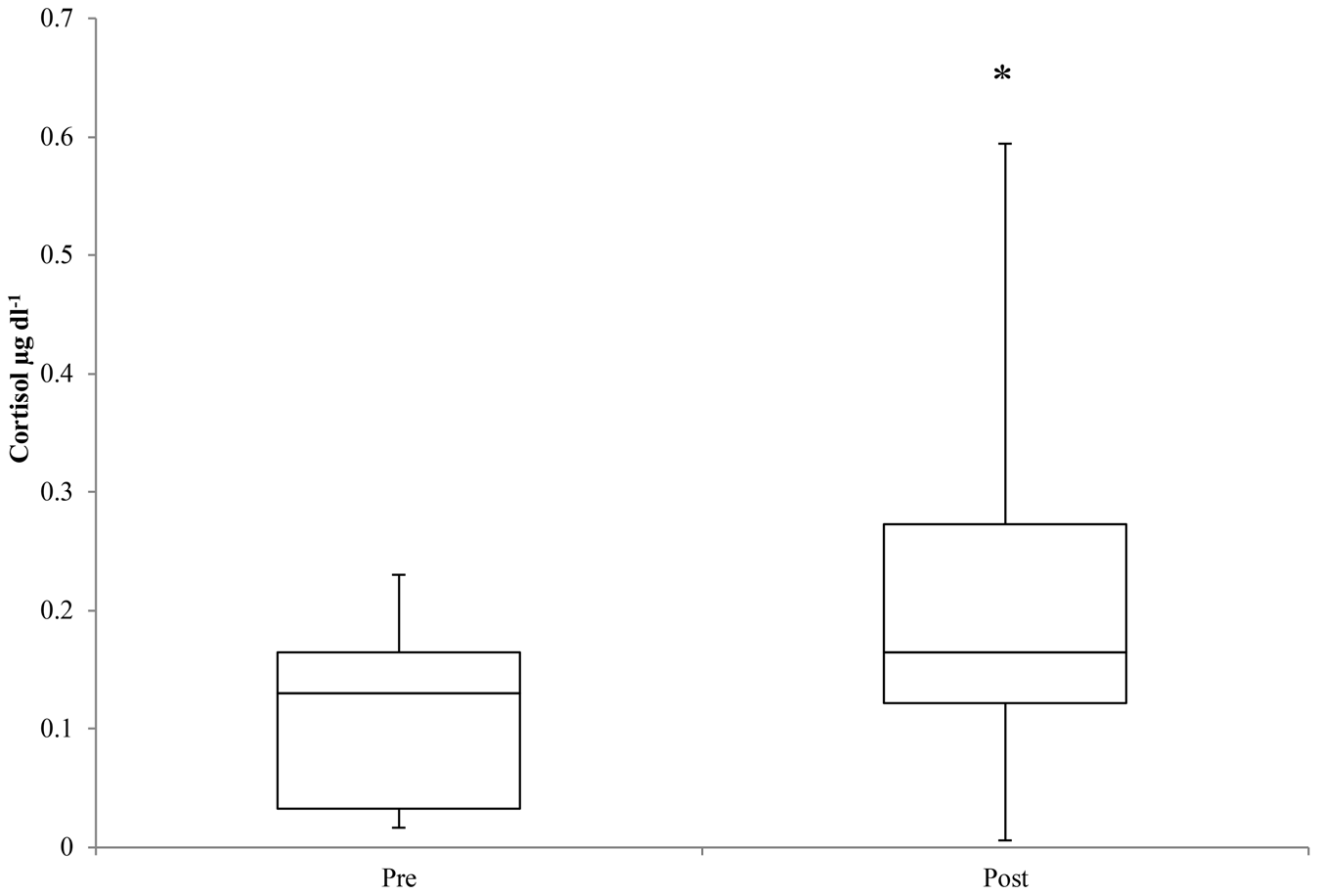
Cortisol concentrations in blow samples collected ‘pre’ and ‘post’ examination for Bristol Bay belugas. A significant increase in blow cortisol was detected, and is indicated by an asterisk (*) (p<0.05).

For belugas from Bristol Bay, cortisol concentrations measured in blow were below 1 µg dl^−1^ and several were lower than the detection limits of the kit (<0.0035 µg dl^−1^). No significant relationship was detected between plasma and blow cortisol for ‘pre’ samples (p>0.05). A significant increase in cortisol values in blow was detected between ‘pre’ and ‘post’ samples for Bristol Bay belugas (p = 0.028) (Figure 9). The magnitude of increase between ‘pre’ and ‘post’ blow samples ranged from an 8.8% increase to a nearly 2000% increase (median  = 109.2%; mean  = 399.2%). A significant positive relationship was detected between handling time (i.e. time from entanglement) and cortisol in both blow (p = 0.019) and plasma (p<0.001). One animal however displayed a large decrease (65%) in cortisol between ‘pre’ and ‘post’ samples in 2013. This animal was actively splashing during restraint and sample collection, and it is suspected that the collection plate became contaminated with water leading to dilution of the cortisol content measured. Overall however, no significant relationship between cortisol content and blow sample volume was detected. While ‘post’ plasma cortisol data was unavailable for 2012, in 2013 there was also a significant increase in plasma cortisol between ‘pre’ and ‘post’ samples (p = 0.003). No significant relationship was detected between plasma cortisol and ‘post’ blow.

## Discussion

The results of this work show that cortisol can be successfully detected in exhaled breath condensate, or blow, from belugas using an EIA, and that blow sampling is a good candidate for monitoring changes in cortisol associated with stressor events in belugas. Such methodology has application for non-invasive, repeated collection of biological information from aquarium animals, with potential for future adaption to free swimming animals which would provide physiological information on animals without the need for capture and restraint.

It is important to note that the depth of the breath (full exhale vs. shallow chuff) seemed to play a role in the volume of sample collected. This is particularly true for aquarium belugas, where 4–8 repeated exhales were required to collect an appreciable volume of sample. When animals were removed from the water and placed upon the exhibit beach during the OWE, their breathing also became shallower accounting for no sample being recovered from several time points. In contrast, wild animals sampled in Bristol Bay, AK performed deep and extended exhales and >50 µl of sample were collected with only 1 or 2 exhales. An explanation for this difference may be that wild animals likely exhale at or near full capacity in order to clear CO_2_ built up from diving or to replenish oxygen stores. The effect on cortisol content should be investigated in future studies. Variation is also seen in recovered condensate volumes from the Bristol Bay animals and this may be due to differences between sample collectors and animal behavior (i.e. remaining still vs. moving head from side to side), and environmental water contamination. Additionally, for Bristol Bay animals, samples collected from animals earlier in the course of a day remained in a cooler for longer periods than later animals and this may have led to greater evaporation before plates were transferred to a freezer.

While statistical significance was not detected, trends in monthly cortisol display a pattern of increased cortisol for 3 animals during fall of 2011, returning to baseline in January and February of 2012. It is interesting to note that a fourth whale was introduced to the beluga habitat at the aquarium in the fall of 2011. Changing social dynamics could have acted as a stressor, resulting in activation of the HPA axis and a rise in circulating cortisol. Increased cortisol in blow was also found for 2 out of 3 animals after 30 minutes out of the water for examination. The differences observed in the magnitude of changes may reflect individual physiology or variability in perception of the stressor which can be shaped by individual experience. Heterogeneity of the variance was also found to be significantly greater at the 30 minute time point as compared with baseline or 24 hours post out of water time points. The increase in the variation of cortisol values suggests increased variation in the neuroendocrine response of individual animals. Such increased variation has been linked with exposure to environmental stressors, such as contaminants, or novel environments [25]. Plasma cortisol however, did not display the same significant increase in variation at the 30 minute time point. This disconnect between blow and blood may be due to timing of sampling in these two matrices and the temporal relationship between changes in blow vs. changes in plasma. More work thus is needed to investigate this relationship and understand both the timing and rate of hormone changes in blood and blow. Variation seen in aquarium animals during the OWE may also reflect other physiological differences related to animal age (∼30 years old vs 9 years old) or sex (2 females vs. 1 male). Indeed, the male, and also youngest animal had a markedly different behavioral response to the OWE, and was the only animal which did not show an increase in blow cortisol.

For wild animals, an increase in blow cortisol was found between ‘pre’ and ‘post’ exam samples despite the inability to standardize sample timing between individuals. The magnitude of change in blow cortisol concentration between ‘pre’ and ‘post’ samples showed some variability, with one animal in particular showing a much larger increase than the rest. The variability in this response may again be explained by individual experience, length of handling time, and modified stressor responses of individual animals due to past exposure to pursuit, boat presence, entanglement or stranding. The animal displaying the largest increase was also the only juvenile female sampled for this study, and thus age and sex may also have influenced the glucocorticoid response. Additionally, no relationship was observed between blood and blow for ‘pre’ samples collected from Bristol Bay animals. We attribute this to the variability in the time of sampling blow in relation to blood during the examination. Cortisol did increase with handling time in both blood and blow samples, indicating that the overall patterns of response were similar.

No direct comparisons of cortisol measurements were made between Bristol Bay belugas and aquarium belugas. Interpretation of such a comparison is likely confounded by multiple factors including the method of collection, daily exposure to stressors not controlled in this study, and also diurnal variation in cortisol secretion. Belugas at Mystic Aquarium were consistently sampled in the morning throughout this study. However, the belugas at Bristol Bay were sampled throughout the course of the day (between 10 am and 4 pm) and thus may have been affected by a diurnal pattern in cortisol secretion (i.e. lower cortisol later in the day) which has been demonstrated in belugas and killer whales [22], [26].

Seasonal variation may also play a role in determining differences in cortisol between Bristol Bay belugas and aquarium animals at Mystic Aquarium. Bristol Bay animals were only sampled in August while monthly sampling occurred in aquarium animals. Though no statistical differences were detected in aquarium animals across months, when data were binned into seasons a significant difference was detected and it is possible that seasonal responses would differ between populations at different latitudes. Significant seasonal changes in cortisol have been reported for mammals in response to large differences in environmental conditions between seasons at high latitude [27], and this may be the case for Bristol Bay animals.

Due to gaps in monthly blood collection no conclusions could be reported as to the relationship between blow and blood cortisol in monthly samples from aquarium animals. However, a significant positive correlation between cortisol values in blow and blood was detected over the course of the OWE, suggesting that blow is a useful alternative matrix for monitoring glucocorticoid responses to stressors. We were able to measure cortisol monthly (baseline conditions) in aquarium belugas, suggesting that cortisol is present in blow even without an obvious stressor event. Establishing normal patterns of variation in these samples could lead to determining a threshold for detecting large deviations in cortisol content indicative of a stress response.

Cortisol measured in blow, however, may reflect only a portion of the total cortisol in plasma. In blood, cortisol can be bound by corticosteroid binding globulin (CBG) or can be free and available to bind with cells and thus exert influence on biological function. Salivary cortisol is thought only to consist of free cortisol [28], [29] which passes to saliva via passive diffusion [5], [30]. If passive diffusion is also the mechanism by which cortisol enters the lungs, blow samples may also contain only the free fraction of cortisol. Both free cortisol and CBG can be measured in blood samples [31], [32], [33], [34] though these analyses were beyond the scope of this study. Variability in the CBG binding should be considered as a possible explanation for lack of a relationship between blow and plasma, and could account for some of the variability observed between individuals in the cortisol values measured in blow during the OWE. Further work should attempt to correlate blow cortisol with the unbound fraction of blood cortisol.

Results of this study demonstrate the ease and efficiency with which blow samples can be obtained from animals within aquaria. Behaviorally, the ability to sample blood from the whales was less consistent than the ability to sample blow during multiple months for all animals, in part due to behavioral challenges. Knowing that blow samples could still be collected, for monitoring hormones or other health parameters when blood cannot be obtained, has important potential within aquaria for both clinical and research applications. For example, the response of cetaceans to changes in their environment or social structure can be monitored in the blow since blood sampling behaviors may be disrupted.

There is no universal standard for a dilution effect in exhaled condensate studies, though the need for one is widely accepted [35]. Urea has been used in human studies, however lack of complete validation of this method and great variability even within subjects has led to some criticism [15], [35], [36], [37]. Attempts to validate urea as a marker of dilution for blow in our laboratory were inconclusive and not reported for this study, though the authors recognize the importance for such work. Samples from Bristol Bay animals displayed a pattern of decreasing cortisol with increasing sample volume, though this relationship was not significant. However, attempting to account for dilution using this method does not account for variation due to individual breaths (e.g. shallow vs. deep exhales), different sample collectors, condensation of water vapor through the respiratory tract, or evaporation on plates collected in the field. While this study aimed to minimize water contamination, accounting for dilution could contribute to explanations for several outlying events noted in this study; 1) the apparent decrease in blow cortisol for animal 3 during the OWE, 2) lower blow cortisol values in some wild animals than in aquarium animals and 3) the single animal from Bristol Bay which showed a large decrease in blow cortisol between pre and post samples. Efforts within our lab continue to investigate potential markers for dilution within blow samples.

Use of blow is a relatively new approach to research and health monitoring, and this study is the first to show that blow can be collected and used to measure cortisol in belugas. Information concerning the health status of populations can be gained by comparing cortisol values in blow samples between healthy populations (e.g. Bristol Bay) and endangered populations (e.g. Cook Inlet).

Due to the small group size used for this study no statistically significant changes in cortisol were detected in blow during the OWE, though an apparent increase was observed in two animals. Statistical analysis of this data however, is likely biased by variability in resting cortisol between individuals. By exploring the heterogeneity of variance at each time point, the 30 min OWE sampling point was determined to be different from baseline suggesting that cortisol levels in response to the stressor were more different between individuals as compared with the mean. Different coping styles among individuals within a population will likely lead to such increased variance in cortisol response, and variance has been suggested as an alternative to analyzing means when looking at the effects of a potential stressor on a population [25]. Thus this approach may also provide important information concerning the impact of stressors on population health.

Collection of blow carries potential for providing a suite of information on the health status of individuals, as it likely also contains proteins, such as cytokines and other markers of health and/or disease. Having this non-invasive approach will be an important tool for research and wildlife conservation and medicine since blow can be sampled more frequently than blood. More work however is needed to better describe the relationship between blood and blow cortisol, and to determine an appropriate dilution control factor. Constraints on this study prevented repetition of the OWE's or use of ACTH challenge, however additional time course studies should be conducted as feasible.

In summary, an appropriate methodology for collecting blow samples from belugas was developed and a commercial EIA validated and used to measure cortisol in beluga blow. This technique provides a means of assessing individual health status without invasive handling and sampling, and is an important tool for monitoring animals in aquaria. While this study applied blow collection to wild belugas under capture and restraint conditions, continuing work will address adaptation of this methodology to free swimming animals without the need for restraint. Blow collection from free swimming animals has been successful in large whales including humpback whales [5] and North Atlantic Right whales [5], [18]. Frere *et al.*, [13] references a pilot study in which blow was successfully collected from wild bottlenose dolphins in Australia, and it is likely that blow can also be obtained from free swimming belugas. Without altering the current collection procedure however, this work provides a means of collecting blow which can be applied to stranded belugas including those of the endangered Cook Inlet population. This approach provides access to physiological data without having to handle the animals.

In the future, the accessibility of this methodology (e.g. ease of obtaining collecting materials) and non-invasive nature of sampling could provide access to free ranging populations, or different species, that are otherwise difficult to handle directly. Future applications of this technique may include monitoring the neuroendocrine response of cetaceans to anthropogenic activities or changing environmental conditions, resulting in health information on threatened or endangered populations.
